# The effect of supplementing CLOSTAT 500 (*Bacillus subtilis* PB6) to yearling steers in a commercial feedyard on health, *Salmonella* spp. prevalence, feedlot growth performance and carcass characteristics

**DOI:** 10.1093/tas/txac131

**Published:** 2022-09-18

**Authors:** Alyssa Word, Paul Rand Broadway, Nicole Burdick-Sanchez, Jeff Carroll, Kristin Hales, Kendall Karr, Ben Holland, Guy Ellis, Casey Maxwell, Landon Canterbury, John Tyler Leonhard, Doug LaFleur, Jerilyn Hergenreder, Sara Trojan

**Affiliations:** Cactus Research, Amarillo, TX 79101, USA; Livestock Issues Research Unit, Lubbock, TX 79403, USA; Livestock Issues Research Unit, Lubbock, TX 79403, USA; Livestock Issues Research Unit, Lubbock, TX 79403, USA; Texas Tech University, Lubbock, TX 79409, USA; Cactus Research, Amarillo, TX 79101, USA; Cactus Research, Amarillo, TX 79101, USA; Cactus Research, Amarillo, TX 79101, USA; Cactus Research, Amarillo, TX 79101, USA; Kemin Industries, Inc., Des Moines, IA 50317, USA; Kemin Industries, Inc., Des Moines, IA 50317, USA; Kemin Industries, Inc., Des Moines, IA 50317, USA; Kemin Industries, Inc., Des Moines, IA 50317, USA; Peak Beef Nutrition and Management Consulting, LLC, Casper, WY 82604, USA

**Keywords:** *Bacillus subtilis*, growth, health, salmonella, steers

## Abstract

British and British × Continental crossbred beef steers, *n* = 2,100; 313 ± 38 kg of initial body weight (BW) were used to evaluate the effects of *Bacillus subtilis PB6* supplementation to yearling steers in a commercial feedyard on health, prevalence of *Salmonella* spp., growth performance, and carcass characteristics. Steers were blocked by arrival date and assigned randomly to pens within the block; pens were randomly assigned to 1 of 2 dietary treatments within block. Treatments, replicated in 15 pens/treatment with 70 steers/pen, included: 1) control (CON), diets containing no supplemental direct-fed microbials; 2) CLOSTAT (CLO), diets supplemented with 0.5 g/steer/d *Bacillus subtilis* PB6 (CLOSTAT 500, Kemin Industries, Des Moines, IA) to provide 6.6 × 10^9^ CFU/g of the active ingredient. Supplementing CLO decreased the overall incidence of morbidity (*P* = 0.03), 10.38% (CLO) vs. 13.43% (CON), decreased the percentage of steers treated once for bovine respiratory disease (BRD; *P* < 0.01), 9.14% (CLO) vs. 12.76% (CON), and decreased the incidence of BRD retreatment (*P* = 0.03) compared with CON. Mortality did not differ among treatments (*P* = 0.23); however, overall deads and removals tended to be less for CLO than CON (53 heads vs. 73 heads respectively, *P* = 0.06). Prevalence of fecal *Salmonella* did not differ among treatments, (*P* ≥ 0.35); overall fecal *Salmonella* counts tended to be less for CLO (1.59 log (10) CFU/g) than CON (2.04 log (10) CFU/g; *P* = 0.07). *Salmonella* concentration in subiliac lymph nodes (n =150/treatment) was not different (*P* = 0.62) between CON (0.22 log (10) CFU/g) or CLO (0.19 log (10) CFU/g); however, there was a 46% reduction in the overall mean prevalence of lymph node *Salmonella* (*P* = 0.46; 15.48% vs. 28.66%) for CLO and CON, respectively. With deads and removals included, final BW was heavier for CLO steers than CON, (654 kg vs. 641 kg, respectively, *P* = 0.05), and average daily gain (ADG; *P* = 0.08) and gain efficiency (G:F; *P* = 0.06) tended to be greater for CLO than CON. With deads and removals excluded, final BW, ADG, and G:F did not differ among treatments (*P ≥* 0.30). Carcass traits were not different between treatments (*P ≥* 0.15). Supplementing CLO throughout the feeding period in a commercial feedyard improved the health outcomes of yearling steers by decreasing BRD and overall treatment rates, reducing the overall abundance of *Salmonella*, and resulting in fewer steers removed from the study compared with CON.

## INTRODUCTION

The beef industry has dedicated decades of research and resources to addressing the economic burden of the bovine respiratory disease (BRD) complex through vaccine technologies, antibiotic therapies, and management; yet, feedyard mortality rates continue to rise, and BRD remains the most economically detrimental disease to the industry (Peel, 2020). The recent estimated direct cost of BRD mortality to the beef industry is $907.8 million, annually, and the average cost for BRD treatment for feedlots is $75 million, with additional losses associated with decreased growth performance and reduced carcass quality (Holland et al., 2010; Peel, 2020). Further, public concerns with antibiotic use in food animal production have led livestock industries to evaluate alternative approaches to disease management (Bencharr et al., 2008). As a result, nutraceutical compounds have come to the forefront of new product development and application in livestock production with implications for health (Ballou et al., 2019). Yet, given the nature of nutraceutical compounds, modes of action are diverse, and health and performance responses to these products are highly variable by compound type and application (Ballou et al., 2019).

The active microbial supplement, *Bacillus subtilis* PB6 (CLOSTAT; Kemin Industries, Des Moines, IA) contains a unique, patented strain of *B. subtilis.* Previous research with *B. subtilis* PB6 supplementation to beef steers has demonstrated positive health outcomes, as Smock et al. (2020a) reported a decrease in BRD treatment rate and decreased overall disease treatment cost. Broadway et al. (2020) indicated that *B. subtilis* PB6 supplementation improved immune responses to a *Salmonella* challenge in Holstein calves and decreased the prevalence of *Salmonella* colonization in small intestinal tissues compared with non-supplemented control calves. In high-producing dairy cows, the rate of cows culled in early lactation was reduced with *B. subtilis* PB6 (Lago et al., 2021). Further, *B. subtilis* PB6 supplementation to transition dairy cattle increased milk production, improved milk:feed ratio, and decreased blood inflammatory markers (Goetz et al., 2022).

The objectives of this study were to evaluate the effects of *B. subtilis* PB6 supplementation on yearling steers in a research commercial feedyard on health, *Salmonella* spp. prevalence, performance, and carcass outcomes. Based-on previous research, our hypothesis was that *B. subtilis* PB6 would result in more favorable health outcomes, lower the incidence of *Salmonella* spp. and improve feedlot growth performance.

## MATERIALS AND METHODS

Study procedures were performed in accordance with the guidelines by the Federation of Animal Science Societies (2020) for the management and use of beef cattle in research.

### Cattle Management and Treatments

British, British × Continental crossbred beef steers (*n* = 2,340) were received at a commercial feedyard in the Texas Panhandle from 14 January 2021 to 20 January 2021. Steers originated from the Texas Panhandle, Alabama, Kentucky, Oklahoma, and Georgia. At arrival processing, steers were weighed individually in a hydraulic chute (Silencer, Moly Manufacturing, Lorriane, KS), and identified individually with duplicate ear tags containing a unique individual ID and feedyard lot number. Management of cattle prior to procurement for the experiment was unknown. For viral respiratory pathogen prevention, steers were vaccinated with Titanium 3 (Elanco Animal Health, Indianapolis, IN), and received an intranasal vaccine, Nasalgen IP, (Merck Animal Health, Madison, NJ). Steers were vaccinated against clostridial diseases with Vison 7 with Spur (Merck Animal Health) and were treated for external parasites with Dectomax (Doramectin, Zoetis, Kalamazoo, MI), and internal parasites with Synanthic (Boehringer Ingelheim Vetmedica, Duluth, GA). Steers were initially implanted with Component TE-IS (80 mg Trenbolone Acetate, 16 mg Estradiol, Elanco Animal Health), and implanted again after 93–98 days on feed with Component TE-200 (200 mg Trenbolone Acetate, 20 mg Estradiol, Elanco Animal Health). Steers selected for study enrollment were within a BW range of ±68.03 kg of each arrival group. Steers were excluded from the study if outside of the targeted BW range, deemed visually ill, lame, or were uncharacteristic of breed type by study personnel. On day 0, pens of steers were weighed on a platform scale prior to delivery of treatment diets to determine initial pen BW, and a 4% shrink was applied (Mettler Toledo, Columbus, OH). The scale is certified once per year and verified once weekly using a loader to ensure that the scale reading variance of East-Middle and Middle-West does not exceed 5%. Verification of the scale is conducted quarterly by Rusty’s Weigh, Lubbock, TX.

Steers enrolled in the study (*n* = 2,100) were blocked by arrival date and BW such that arrival dates were represented equally within each BW block. A computer-generated randomization schedule was used to assign eligible steers to pens within block. Within each group of two eligible animals in the chute at processing, one was randomly assigned to each pen (70 steers/pen) in the block. Within blocks, pens were assigned randomly to one of two dietary treatments, thus, treatments were replicated in 15 pens. Steers were allocated into 30 contiguous, open-air, soil surface pens (53.34 m × 18.29 m) with a minimum of 13.9 m^2^/animal and a minimum of 25.4 cm of linear bunk space. Treatments, arranged in a randomized complete block design included: 1) control (CON), diets contained no supplemental bacterial or yeast direct-fed microbials and were without *Bacillus subtilis* PB6; 2) CLOSTAT (CLO), diets supplemented with 0.5 g/hd/d *Bacillus subtilis* PB6 (CLOSTAT 500) to provide 6.6 × 10^9^ CFU/g of active ingredient. CLOSTAT 500 was added to the starter and finisher rations through a micro-ingredient machine maintained by Micro Technologies (Amarillo, TX); briefly, CLO was added with water to the Roto-Mix truck (Roto Mix, Dodge City, KS) via the micro-ingredient machine, and the diet was allowed to mix for at least 3 min following the addition of CLO.

### Feeding and Health Management

Following arrival processing, steers were fed a complete starter feed, RAMP (Cargill Corn Milling, Bovina, TX). Loose hay was top-dressed to the starter feed for at least 3 d following arrival. Steers were transitioned to the finishing ration by a two-ration system by which 10%–5% of the daily feed call of RAMP was replaced with the finishing ration. Incremental changes to the ration were made every 2–4 d. Feed additives, Monensin (Rumensin, Elanco Animal Health), 20 g/ton, and an antimicrobial for liver abscess prevention, Tylosin (Tylan, Elanco Animal Health), 10 g/ton, were included in the RAMP starter feed. The steam flaked corn-based finishing ration (Table 1) was prepared at the feed mill on-site and included a vitamin-trace mineral supplement to exceed NASEM (2016) recommendations as well as feed additives, including an ionophore, Monensin, 42.1 g/ton, and Tylosin, 7.5 g/ton, were included in the finishing diet for the duration of the experiment. Ractopamine-hydrochloride was administered the final 31 days on feed (Optaflexx, Ractopamine-hydrochloride, Elanco Animal Health) through the micro-ingredient machine. Ingredient and nutrient composition of the finishing ration are in Table 1.

**Table 1.** Ingredient and nutrient composition of starting and finishing rations.

Ingredient, % of dry matter	Dietary treatment^1^	
	CON	CLO
Starting ration		
RAMP^2^	100	99.98
Micro-ingredients	**--**	0.02
CLOSTAT^®^ 500^3^, g/T	**--**	0.50
Composition, % of dry matter^2^		
NE_m_, Mcal/kg	2.11	2.11
NE_g_, Mcal/kg	1.41	1.41
Crude protein, %^4^	21.5	21.3
Calcium, %^4^	1.53	1.66
Phosphorus, %^4^	0.82	0.39
Finishing ration		
Steam flaked corn	53.67	53.63
Wet distiller’s grains	19.15	19.16
Sweet bran plus^4^	18.34	18.37
Ground corn stalks	7.45	7.44
Yellow grease	1.36	1.37
Micro-ingredients^5^	0.03	0.03
CLOSTAT^®^ 500^3^	–	0.50
Composition, % of dry matter		
vNE_m_, Mcal/kg^6^	2.17	2.17
NE_g_, Mcal/kg^6^	1.45	1.45
Crude protein, %^4^	14.7	14.8
Calcium, %^4^	0.79	0.82
Phosphorus, %^4^	0.51	0.51

^1^CON = control; CLO = CLOSTAT steers fed control diet supplemented with 0.5 g/hg/d *Bacillus subtilis* PB6(CLOSTAT 500, Kemin Industries, Des Moines, IA).

^2^RAMP = commercially manufactured complete starter feed (Cargill Corn Milling, Dalhart, TX), including minerals, vitamins,20 g/T monensin (Rumensin 90, Elanco Animal Health, Greenfield, IN) and 10 g/T tylsoin (Tylan, Elanco Animal Health); energy values from Cargill Corn Milling.

^3^CLOSTAT 500, Kemin Industries, delivered through micro-ingredient machine, containing 6.6 × 10^9^ CFU *Bacillus subtilis* PB6.

^4^Analysis by Servi-Tech Laboratories, Amarillo, TX.

^5^Micro-ingredients for finishing diet included minerals, vitamins, 42 g/T monensin (Rumensin 90, Elanco Animal Health), 7.5 g/T tylosin (Tylan, Elanco Animal Health), and Ractopamine-hydrochloride, 27.3 g/T the final 31 days on feed (Optaflexx, Elanco Animal Health).

^6^Formulated values based on NASEM (2016).

Feed bunks were evaluated visually at approximately 0600 h, 1800 h, and 2100 h for the presence of residual feed. The observation at 0600 h was the primary check, and reactions and behavior of steers at the time of feeding were assessed and used to determine the daily feed call. Feed calls were made to provide feed to appetite and to minimize feed carry-over; large increases or decreases in daily feed calls were avoided if possible. Feed was delivered three-times daily using a truck mounted with a Roto-Mix delivery box and recorded to the nearest 4.5 kg. Samples of each ration were collected daily from the feed bunk, and subsamples of each collection were dried at 100 °C to determine the dry matter of the ration. Daily dry matters were averaged weekly and used to calculate dry matter intake.

### Health

Steers were monitored daily for health abnormalities. Unusual observations were recorded by exception. Animals exhibiting signs of BRD, digestive disturbance, lameness, injury, or other malady were taken to a hospital facility for further evaluation, and treatment was performed, if necessary, according to the Feedyard treatment protocol. Nuflor (Merck Animal Health) was administered to steers requiring the first treatment for BRD per labelled route and dose; Advocin (Zoetis) was administered to steers requiring a second treatment for BRD per labelled route and dose. If treatment for BRD was required, and the projected shipment for slaughter was less than 50 d, Advocin was administered as the treatment for BRD, regardless of first or second treatment status. For steers removed from a pen for suspected BRD but with a rectal temperature <40 °C, a chalk mark was placed on their back, and the animal was diagnosed as a “respiratory observe” without receiving BRD treatment.

Animals were removed from the study if deemed as not growing at the same rate as pen-mates, non-responsive to BRD treatment, injured, or identified as a buller. Date, the reason for removal, and BW were recorded. A field necropsy was performed on all animals that died or were euthanized, and the date of death, and diagnosis, when determinable was recorded. Overall, 69 steers were removed from the study.

### Determination of Salmonella Prevalence

Fecal samples were aseptically collected, preventing soil contamination, via convenience grab sampling from each pen in 45 d increments throughout the duration of the experiment. Fresh fecal matter was collected from at least 10 animals/pen from multiple locations throughout the pen and composited by pen at each collection day, *n* = 15 pens/treatment for each day of collection. Samples were obtained in the months of January through September, encompassing winter, spring and summer seasons. Pen conditions at collection ranged from muddy to dry; collection on d 45 took place one week after a major winter storm. Fecal samples from each pen were analyzed in duplicate, and samples were processed in a similar manner as described by Broadway et al. (2020). Briefly, 25 g of pooled sample was weighed, and homogenized in 1:10 PBS for 2 min in a stomacher. A 1 mL aliquot was removed from the homogenate and subjected to spiral plating (Eddy Jet 2W; IUL Instruments) on Brilliant Green Agar with Novobiocin. Following overnight incubation at 37 °C, colonies were imaged, and phenotypical colonies were counted (Sphere Flash; IUL Instruments) to determine *Salmonella* concentrations defined as colony forming units per g accounting for a dilution factor. Additional aliquots were enriched in Tetrathionate Broth with Iodine and Rappaport Vassiladus enrichment broths and incubated overnight at 37 °C and 42 °C, respectively. Enrichments were streak plated onto Brilliant Green and Xylose Lysine Tergitol-4 agars. Plates were incubated overnight at 37 °C prior to observation for phenotypical colonies. Phenotypic colonies were subjected to a *Salmonella* latex agglutination text (Oxoid) for confirmation. Subiliac lymph nodes were obtained from a subset of carcasses within each lot at harvest; overall, *n* = 150 nodes/treatment were collected for analysis. Samples were processed similarly to that described by Arthur et al. (2008), and quantification and isolation were performed as described previously.

### Days on Feed and Carcass Data Collection

Cattle were projected for days on feed as assigned by the feedyard projection program. Optaflexx (Ractopamine-hydrochloride, Elanco Animal Health) was administered for the final 31 days on feed at the rate of 27.3 g/T. Prior to beta-agonist administration, steers were visually evaluated for condition and BW and determination of shipment week by the General Manager of the feedyard. Steers within respective weight blocks were transported 104 km to a commercial abattoir on 26 July, 2, 9, 16, 23, 30 August, and 6 September 2021. For each slaughter date, steers were shipped in the morning with pen weight measurements obtained prior to shipping. Final pen weights were by shrunk 4% to determine the final BW.

Carcass data were collected by trained personnel from the Beef Carcass Research Center (West Texas A&M University, Canyon, TX). The individual animal ear tag number in the sequence of the harvest was recorded and affixed to a harvest sequence number for each carcass. Plant carcass ID and hot carcass weight (HCW) were recorded and verified by carcass sequence number. Livers were scored for the presence and severity of abscesses on the scale of none, A, A−, A+, and for the presence of other abnormalities. Lungs were scored for severity of consolidation (1 = 0–15%, 2 = 15–50%, 3 = >50%), severity of pleural adhesions (minor or extensive), and for presence of other abnormalities.

Carcasses were graded after approximately 36 h chill; the USDA quality grade was determined by verified USDA personnel and the yield grade was captured by packing plant camera data and verified by USDA personnel. The HCW and camera grading measurements were obtained from packing plant records. The dressing percentage for each pen was calculated as the mean HCW/ mean shrunk live BW × 100, pen final BW shrink = 4%.

### Statistical Analysis

Growth performance was calculated both on a deads and removals-included and excluded basis. Data were analyzed as a complete block design using ANOVA (Stata 17; Statacorp, College Station, TX) with a pen as the experimental unit. The model included the fixed effects of treatment and block. Categorical data (removals, mortality, carcass grade distributions, and liver and lung scores) were analyzed using logistic regression (binreg; Stata 17), with treatment and block included in the model as fixed effects.

*Salmonella* counts were determined based on mean plate counts and dilution factor were log-transformed to achieve normality and analyzed using the PROC GLIMMIX procedure of SAS (SAS v 9.4, SAS Inst. Inc., Cary, NC) with the fixed effects of treatment, time, and the interaction. The pen was utilized as the experimental unit, and quantification data are reported as log CFU/g. Prevalence data were analyzed as binomial proportions in PROC GLIMMIX of SAS and reported as a percentage of positive samples within each treatment. For all data, *α* = 0.05, and *P*-values between 0.05 and 0.10 were discussed as trends.

## RESULTS AND DISCUSSION

### Animal Health

Data for animal health are summarized in Table 2. The overall rate of CLO steers treated for any health reason was 29% less than CON (*P* = 0.03). There was a 39.6% decrease in the rate of steers treated once for BRD for CLO steers compared with CON (*P* < 0.01), and the percentage of steers requiring a second BRD treatment was less for CLO than CON (2.10% vs. 3.33%, respectively; *P* = 0.03). The rectal temperature at first treatment did not differ among treatments (*P* = 0.19). There was no difference between treatments in the rate of steers removed from the pen for a suspected respiratory disease but with rectal temperature ≤40 °C (*P* = 0.64).

**Table 2.** Animal accountability, morbidity, and mortality of steers fed 0.5 g/steer/day *Bacillus subtilis*PB6 (CLOSTAT 500) compared with non-supplemented control steers.

Item	Dietary treatment^1^		*P-*value
	CON	CLO	
Day 0 steers, *n*	1,050	1,050	–
*Morbidity*			
Total first pull, *n* (%)	141 (13.43)	109 (10.38)	0.03
Acute interstitial pneumonia	1 (0.10)	4 (0.38)	–
Bloat	3 (0.29)	1 (0.10)	–
Buller	1 (0.10)	1 (0.10)	–
Lame	5 (0.48)	5 (0.48)	–
vOther^2^	9 (0.86)	6 (0.57)	–
Pneumonia	122 (11.62)	91 (8.67)	–
Prolapse	0 (0.00)	1 (0.10)	–
Respiratory morbidity			
1st treat, %	134 (12.76)	96 (9.14)	<0.01
2nd treat, %	35 (3.33)	22 (2.10)	0.03
Respiratory Observe^3^, *n* (%)	1.90	2.19	0.64
Temperature, 1st treatment, °C	40.44	40.38	0.19
Mortality, *n* (%)	33 (3.14)	24 (2.29)	0.23
Digestive, *n*	9 (0.86)	6 (0.57)	0.44
Other^2^	4 (0.38)	5 (0.48)	0.74
Respiratory, *n*	20 (1.90)	13 (1.24)	0.22
Removed, *n* (%)	40 (3.18)	29 (2.76)	0.18
Buller, *n*	2 (0.19)	0 (0.00)	0.99
Digestive, *n*	1 (0.10)	0 (0.00)	0.99
Other, *n*	16 (1.52)	15 (1.43)	0.86
Respiratory, *n*	21 (2.00)	14 (1.33)	0.23
Total outs, *n* (%)	73 (6.95)	53 (5.05)	0.06
Shipped, *n*	977	997	–

^1^CON = control, standard finishing diet; CLO = CLOSTAT steers fed control diet supplemented with 0.5 g/hg/d *Bacillus subtilis* PB6(CLOSTAT 500, Kemin Industries, Des Moines, IA), administered through the micro-ingredient machine.

^2^“Other”, diagnosis includes lameness, rectal prolapse, hardware disease or heart failure, peritonitis, or enteritis.

^3^Describes cattle removed from pen for suspected respiratory disease but with rectal temperature ≤40 °C.

Dietary treatment did not affect overall mortality (2.29% or 3.14% for CLO and CON, respectively; *P* = 0.23). Likewise, mortality diagnosis did not differ among treatments (*P* ≥ 0.22). The overall percentage of CLO steers removed from the study was not different from CON (2.76% vs. 3.81%, respectively; *P* = 0.18). However, total outs (the combined percentage of dead and removed steers) tended to be less for CLO than CON (53 steers vs. 73 steers; *P* = 0.06).

Previous literature with *B. subtilis* PB6 supplementation in cattle support these findings. Smock et al. (2020a) supplemented *B. subtilis* PB6 to high-risk feeder steers with 34.6% respiratory morbidity after arrival metaphylaxis and noted a 9.3% decrease in the number of steers treated initially for BRD, and a trend for an 11.5% decrease in steers re-treated for BRD for *B. subtilis* PB6 supplementation over control. Authors projected a cost savings of $3.95 per steer for antimicrobial cost alone for *B. subtilis* PB6 supplemented steers when compared to control. Although not significant, single-sourced, low-stress feedlot steers supplemented with *B. subtilis* PB6 had a 33% decrease in the first BRD treatment pull rate compared with control (Wilson et al., 2019). Further, in high-producing dairy cows, there were fewer cows culled during early lactation when supplemented with *B. subtilis* PB6 than non-supplemented control cows (Lago et al., 2021).

The microbial communities of the lung are exposed to intestinal bacterial populations and their metabolites through the mesenteric lymph system, as such, positive associations with respiratory health and lung exposure to commensal bacterial populations have been noted in human health (Allie et al., 2019; Enaud et al., 2020; Gollwitzer et al., 2014). Moreover, microbial communities in the respiratory tract of cattle vary during the transition of weaning to the early feeding period (Timsit et al., 2020). The reduction in respiratory illness observed in this study and across other research with *B.subtilis* PB6 (Wilson et al., 2019 and Smock, et al., 2020a) is likely attributed to its role in maintaining the health and stability of intestinal commensal bacterial populations.

### Growth Performance

#### Dead and Removed Steers Included.

The growth performance data are provided in Table 3. By experimental design, initial BW did not differ among treatments (*P* = 0.71). Dry matter intake throughout the experimental period was not different between treatments (*P* = 0.14). With dead and removed steers included in the performance analysis, days on feed tended to be greater for CLO than CON (*P* = 0.09); final BW was 13 kg heavier for CLO supplemented steers than CON (*P* = 0.052), and CLO steers gained 14 kg more throughout the study duration than CON (*P* = 0.046). Average daily gain tended to be greater for steers in the CLO treatment than those in CON (*P* = 0.08), and there was a trend for a 2.91% improvement in G:F for CLO vs. CON (*P* = 0.06).

**Table 3.** Live growth performance of steers fed *Bacillus subtilis*PB6 (CLOSTAT 500) compared with non-supplemented control steers.

Item	Dietary treatment^1^		SEM	*P-*value
	CON	CLO		
Pens, n	15	15	–	–
Steers, n	1,050	1,050	–	–
Initial BW, kg^2^.	308	309	0.82	0.71
Dry matter intake, kg.	10.05	10.12	0.04	0.14
*Live performance with dead and removed steers included*				
Days on feed	196	198	0.97	0.09
Final BW, kg	641	654	4.47	0.052
Total BW gain, kg	332	346	4.20	0.046
Average daily gain, kg	1.70	1.74	0.017	0.08
Gain:feed	0.167	0.172	0.002	0.06
*Live performance with dead and removed steers excluded*				
Days on feed	205	205	--	--
Final BW, kg.	675	677	1.60	0.29
Total BW gain, kg.	367	369	1.44	0.33
Average daily gain, kg.	1.79	1.80	0.01	0.37
Gain:feed	0.176	0.178	0.001	0.30

^1^CON = control, standard finishing diet; CLO = CLOSTAT steers fed control diet supplemented with 0.5 g/hg/d *Bacillus subtilis* PB6(CLOSTAT 500, Kemin Industries, Des Moines, IA), administered through the micro-ingredient machine.

^2^Inital body weight x 0.96.

#### Dead and removed steers excluded.

 There was no difference in final BW, total weight gain, ADG or G:F among treatment groups when data were analyzed with the dead and removed steers excluded (*P* ≥ 0.29; Table 3). These data are consistent with results reported in the literature with *B. subtilis* PB6 supplementation throughout the feeding period. Cumulative feedlot ADG, DMI or G:F was not affected by *B. subtilis* PB6 supplementation throughout the feeding period in high-risk feeder steers, but did improve ADG by 13.22% and increased DM intake, 0.42 kg, during the 56-d receiving period over the control group (Smock et al., 2020a). In single-sourced calves, G:F tended to be greater for *B. subtilis* PB6 supplemented steers than for control; although other growth performance parameters were not different (Wilson et al., 2019). Further, feedlot growth performance was not altered by *B. subtilis* PB6 supplementation throughout the feeding period in single source yearling steers with minimal environmental stress and a low pathogen prevalence (Smith et al., 2021). In *E.coli* challenged broilers, supplementing *B. subtilis* PB6 increased ADG and feed efficiency over control (Teo and Tan, 2006).

Analysis of growth performance data on a “deads-out” basis allows the biology of cattle productivity to be more clearly and accurately expressed (Stegner et al., 2013); however, accounting for the inherent financial loss associated with unrecoverable feed costs and lost performance of dead and removed cattle represent significant economic consequences to cattle feeding operations. In the present study, the results for growth performance on a “deads-in” basis encumbered the poor-performance associated with the greater population of cattle treated in the CON group than CLO. Moreover, there were 20 more steers from the CLO treatment than the CON treatment that were eligible to be sold without an alternate marketing structure or that represented an entire financial loss.

To understand the economic impact of the dead and removed cattle by treatment in the present study, the head count by treatment for railer categories provided in Table 2, and the cutter cow prices derived from the USDA National Weekly Cow and Bull Report for the week of 08/05/2022 were used. Values for carcass weight from the report were converted to live weight using a 48% standardized dressing percentage, and the live weight of railers was applied to appropriate weight categories from the report and revenue was determined as such. The price of live-fed cattle from the week of 08/05/2022 from the USDA Daily Direct Steer and Heifer Slaughter Cattle Summary was used to value fed cattle sold. The overall revenue (railers + fed cattle) for the CLO treatment was $57,676 greater than the CON treatment, illustrating the financial burden of death loss and railer cattle. The overall cost of CLO for the feeding period was $10,395, representing a 5.55:1 return on investment advantage for CLO compared with CON.

### Post-Harvest Characteristics

#### Carcass characteristics.

 The analysis of carcass characteristics is provided in Table 4. There were 25 more carcasses accounted for in the CLO treatment than CON (*P* = 0.05), aside from the dead and removed steers (*n* = 20), it is unknown why the additional five carcasses were not available for evaluation. Hot carcass weight did not differ among treatments (*P* = 0.37). Carcass gain, estimated from the assumption of 58% initial dressing percentage, was 11 kg greater for CLO supplemented steers than CON with deads and removals included in the analysis (*P* = 0.03), but not different when analyzed with deads and removals excluded (*P* = 0.43). Dressing percentage, longissimus muscle area, marbling score, 12th rib-fat thickness, and distributions of quality and yield grades were similar between CON and CLO treatments (*P ≥* 0.15). Results for carcass characteristics by feeding *B. subtilis* PB6 throughout the feeding period in the aforementioned experiments corroborate the findings for carcass parameters in this study (Smith et al., 2021; Smock et al., 2020a; Wilson et al., 2019).

**Table 4.** Carcass weight and characteristics of steers fed *Bacillus subtilis*PB6 (CLOSTAT 500) compared with non-supplemented control steers.

Item	Dietary treatment^1^		SEM	*P*-value
	CON	CLO		
Carcasses^2^, *n*	972	997	0.56	0.05
Hot carcass weight, kg	434	435	0.93	0.37
Hot carcass weight gain, DRI, kg^3^	216	227	3.28	0.03
Hot carcass weight gain, DRO, kg^4^	255	256	0.84	0.43
Dressing percentage, %	64.32	64.27	0.08	0.65
Longissimus muscle area, cm^2^	96.23	96.44	0.29	0.62
Marbling score^5^	507	502	4.40	0.41
Fat thickness, cm	1.72	1.74	0.02	0.48
USDA quality grade distribution				
Prime, %	5.58	6.77	--	0.28
Choice, %	75.92	73.19	--	0.15
Select, %	18.40	19.33	--	0.55
No roll, %	0.10	0.21	--	0.54
Commercial, %	0.00	0.51	--	0.99
USDA yield grade distribution				
YG 1, %	4.18	3.79	--	0.63
YG 2, %	28.27	27.71	--	0.76
YG 3, %	44.01	43.03	--	0.70
YG 4, %	20.53	21.73	--	0.52
YG 5, %	3.01	3.74	--	0.37

^1^CON = control, standard finishing diet; CLO = CLOSTAT steers fed control diet supplemented with 0.5 g/hg/d *Bacillus subtilis* PB6(CLOSTAT 500, Kemin Industries, Des Moines, IA), administered through the micro-ingredient machine.

^2^Number of carcasses analyzed after reconciliation between carcass data collection service and packing plant data. Carcasses from which lot number could not be confirmed, USDA condemned, or carcasses reported as “could not be tracked through harvest floor”, were not analyzed.

^3^Hot carcass weight gain with dead and removals included (DRI); calculated from the assumption of 58% initial dressing percentage.

^4^Hot carcass weight gain with dead and removals omitted (DRO); calculated from the assumption of 58% initial dressing percentage.

^5^300 = slight 0; 400 = small 0; 500 = modest 0.

#### Liver and lung characteristics.

 Liver and lung characteristics are summarized in Table 5. The percentage of edible livers did not differ between treatments (*P* = 0.76), and the rate of abscessed livers and severity did not differ by treatment (*P* = 0.90). There was no difference between CON and CLO for liver scores (*P* ≥ 0.11) or for livers identified with abnormal ailments (*P* ≥ 0.15). Lung consolidation rates were not different between CON and CLO supplemented steers (*P* ≥ 0.54), and other measured lung parameters did not differ between treatments (*P* ≥ 0.13). These findings agree with Smock et al. (2020a) and Smith et al. (2021) as these experiments also did not denote a difference in the incidence of liver abscesses or severity or lung characteristics (Smock et al., 2020a) with *B. subtilis* PB6 supplementation compared with control.

**Table 5.** Liver and lung scores of steers fed 0.5 g/steer/day *Bacillus subtilis*PB6 (CLOSTAT 500) compared with non-supplemented control steers.

Item	Dietary treatment^1^		*P-*value
	CON	CLO	
Livers, *n*	972	997	
Liver abscess scores^2^			
Edible, %	80.04	79.47	0.76
Abscessed, %	8.69	8.54	0.90
Abscess severity distribution			
A−, %	4.13	4.88	0.43
A, %	1.06	0.40	0.11
A+, %	3.50	3.27	0.74
Lungs, *n*	954	990	–
Lung consolidation score^3^			
1, %	17.82	16.76	0.56
2, %	13.76	12.64	0.54
3, %	7.78	7.92	0.57
Lung fibrin score			
Minor, %	14.51	13.31	0.44
Extensive, %	14.82	12.40	0.13
Lung status			
Normal, %	32.30	34.97	0.18
Abnormal, %	46.22	43.04	0.18
Condemned, %	21.38	21.68	0.99
Inflated, %	0.31	0.62	0.41

^1^CON = control, standard finishing diet; CLO = CLOSTAT steers fed control diet supplemented with 0.5 g/hg/d *Bacillus subtilis* PB6(CLOSTAT500, Kemin Industries, Des Moines, IA), administered through the micro-ingredient machine.

^2^Severity of liver abscesses scoring system was the following: A− (one or two small abscesses); A (two to four well-developed abscesses, less than 2.54 cm diameter); A+ (one or more large active abscesses, greater than 2.54 cm in diameter with inflammation of surrounding tissues.

^3^Lung consolidation scores include: 1 = 0–15% consolidation of tissue; 2 = 15–50% consolidation of tissue; 3 = >50% consolidation or tissue or portion of lung missing.

### Prevalence of Salmonella

**Figure 1.**
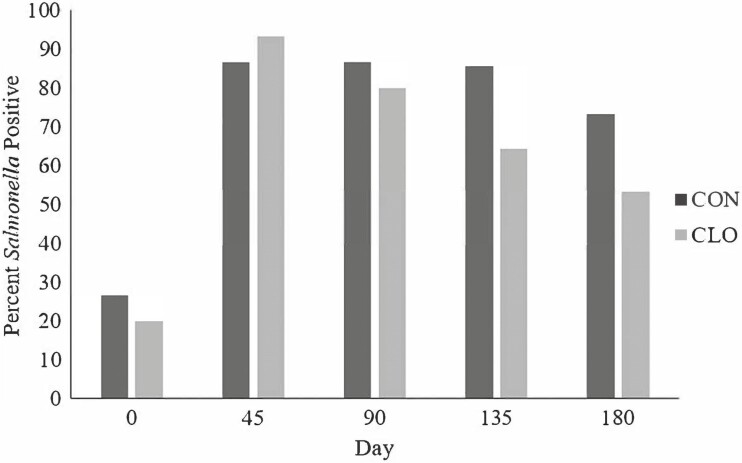
Fecal *Salmonella* prevalence, percentage of total pens within treatment, of feedlot cattle sampled throughout the feeding period supplemented with 0.5 g/hd per day of *Bacillus subtilis* PB6, CLOSTAT 500, Kemin Industries, Des Moines, IA, (CLO) or not (CON). Fresh fecal samples were collected from at least 10 animals per pen and composited by pen on each collection day, *n* = 15 pens/treatment for each day of collection. Samples were collected the months of January–September, and were obtained in 45 day increments throughout the duration of the experiment. Treatment: *P* = 0.35, Day: *P* < 0.01, Treatment * Day: *P* = 0.76; SEM = 5.4.

**Figure 2.**
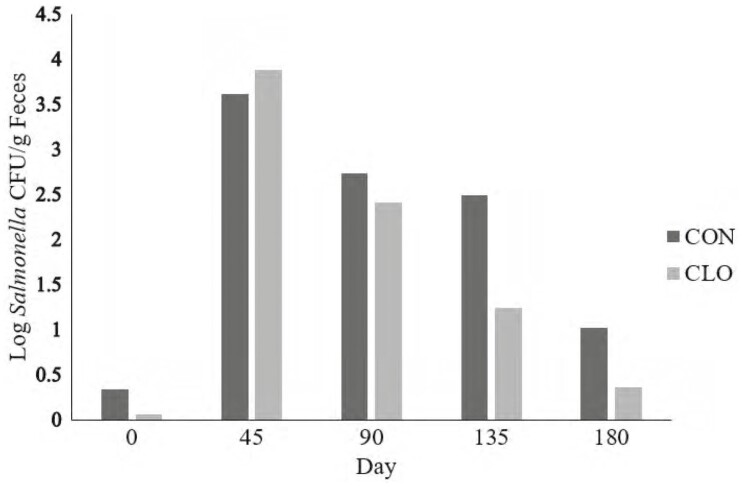
Fecal *Salmonella* concentrations from feedlot cattle sampled throughout the feeding period supplemented with 0.5 g/hd per day *Bacillus subtilis* PB6, CLOSTAT 500, Kemin Industries, Des Moines, IA, (CLO) or not (CON). Fresh fecal samples were collected from at least 10 animals per pen and composited by pen on each collection day, *n* = 15 pens/treatment for each day of collection. The experiment took place the months of January–September, and samples were obtained in 45 day increments throughout the duration of the experiment. Treatment: *P* = 0.07, Day: *P* < 0.01, Treatment * Day: *P* = 0.3; SEM = 0.3.

No differences (*P* ≥ 0.35) were observed in fecal *Salmonella* prevalence between CON and CLO cattle. However, there was a difference in fecal *Salmonella* prevalence and quantity across sampling days (*P* < 0.01; Figures 1 and 2). Upon arrival, fecal *Salmonella* prevalence was 26.7% and 20% for CON and CLO respectively; however, by d 45 the percentage of prevalence positive pens elevated to 86% and 93% for CON and CLO, respectively. While the seasonality of *Salmonella* has been explored, and suggests that *Salmonella* may be more prevalent in warmer months (Edrington et al. 2004), the sampling that occurred on day 45 was in close proximity to a winter storm. Therefore, these differences may be partially explained by increased exposure to environmental *Salmonella* within the pens juxtaposed with the added stress and precipitation from a winter storm. Although the storm was significant, there was no major disruption to cattle feeding or health management as a result of the weather event; cattle had continuous access to water and maintained the prescribed feeding schedule. There was a tendency (*P* = 0.07) for overall mean fecal *Salmonella* concentration to be decreased in CLO (1.59 log CFU/g) compared with CON (2.04 log CFU/g; Figure 2). Similarly, there was a day effect for fecal *Salmonella* concentration (*P* < 0.01) by which *Salmonella* concentrations were very few upon arrival but spiked to over 3.5 log CFU/g by day 45. Each subsequent collection from days 45 to 180 yielded a slight decrease in overall fecal *Salmonella* concentration. Fecal *Salmonella* concentrations were numerically reduced in CLO steers on all days with the exception of day 45 (*P* = 0.59; 3.62 vs. 3.88 log CFU/g for CON and CLO, respectively, data not shown). The largest difference between treatments in fecal *Salmonella* concentrations occurred on day 135 (*P* = 0.01; 2.50 vs. 1.23 log CFU/g for CON and CLO, respectively, data not shown).

Previous data reported that *Bacillus subtilis* PB6 decreased *Salmonella* in gastrointestinal tissues during an experimental *Salmonella* challenge (Broadway et al., 2020). Similarly, Smock et al. (2020b) reported a large decrease in fecal *Salmonella* concentrations in feedlot steers supplemented with *B. subtilis* PB6 compared with non-supplemented steers. Yet, Smith et al. (2021) reported no differences in fecal *Salmonella* when supplementing *B. subtilis* PB6 to yearling feedlot steers; however, *Salmonella* was isolated from only a small percentage of the fecal samples collected.

**Figure 3.**
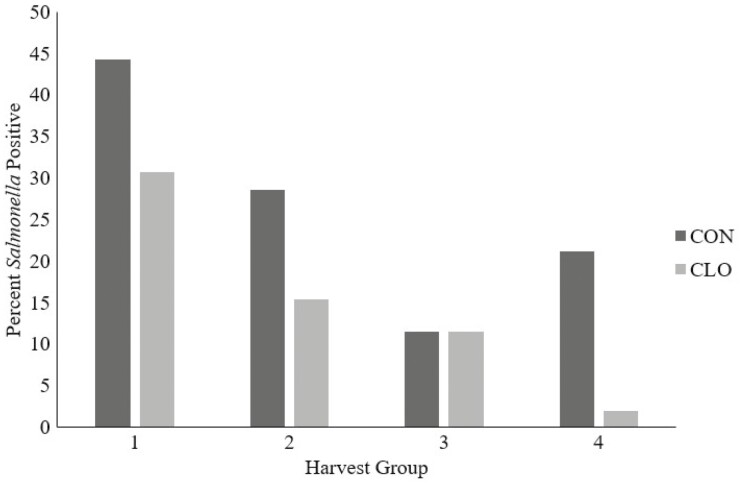
Lymph node *Salmonella* prevalence from feedlot cattle sampled across four harvest dates that were supplemented with 0.5 g/hd per day *Bacillus subtilis* PB6, CLOSTAT 500, Kemin Industries, Des Moines, IA, (CLO) or not (CON). Lymph nodes were collected across multiple harvest dates representing *n* =150 nodes/treatment. Treatment: *P* = 0.45, Day: *P* = 0.16, Treatment * Day: *P* = 0.93, SEM=18.68.

**Figure 4.**
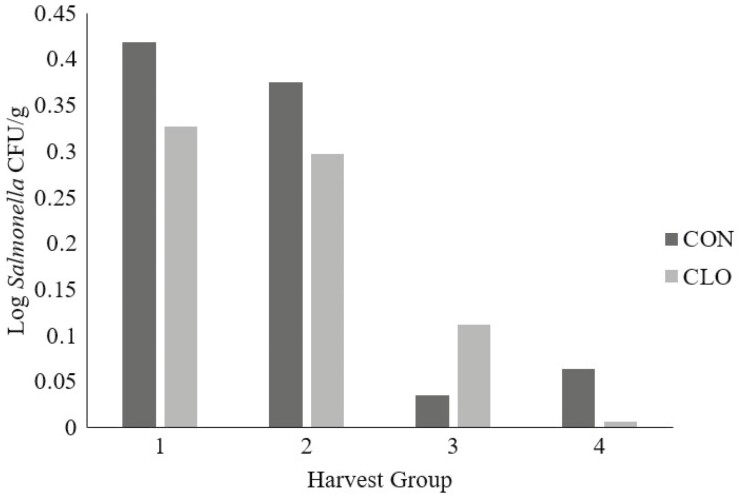
Lymph node *Salmonella* concentrations from feedlot cattle sampled across four harvest dates that were supplemented with 0.5 g/hd per day *Bacillus subtilis* PB6, CLOSTAT 500, Kemin Industries, Des Moines, IA, (CLO) or not (CON). Lymph nodes were collected across multiple harvest dates representing *n* =150 nodes/treatment. Treatment: *P* = 0.62, Day: *P* < 0.01, Treatment * Day: *P* = 0.87.

Subiliac lymph nodes were collected at harvest. *Salmonella* prevalence did not differ by treatment in subiliac lymph nodes (*P* = 0.46; Figure 3); however, there was a 46% decrease in the overall mean lymph node *Salmonella* prevalence (28.66% vs. 15.48% for CON and CLO, respectively). There was no difference (*P* = 0.16) for lymph node *Salmonella* prevalence between harvest dates. There was no difference (*P* = 0.62) between CON (0.22 log CFU/g) and CLO (0.19 log CFU/g) when evaluating *Salmonella* concentrations within the lymph nodes (Figure 4). Steers were harvested on 4 separate days, and there was a difference in lymph node *Salmonella* concentrations across harvest days (*P* < 0.01).

Contamination of musculoskeletal lymph nodes with pathogens has become a topic of concern as these lymph nodes are generally incorporated into grinds and/or contribute to cross-contamination. Additionally, topical pathogen reduction interventions may be ineffective in mitigating pathogens such as *Salmonella* that are harbored in peripheral lymph nodes. Gragg et al. (2013) reported in a nationwide surveillance project that ~10% of subiliac lymph nodes from feedlot cattle harbored *Salmonella,* among presumably healthy animals. The results of Gragg et al. (2013) are slightly less than this study; however, cattle were sampled across seasons and different regions across the U.S. Broadway et al. (2020) reported no difference in *Salmonella* concentrations in peripheral lymph nodes in Holstein calves following an experimental challenge in which calves were supplemented with *B. subtilis* PB6 after being inoculated with *Salmonella typhimurium*. In a feedlot trial conducted by Smith et al. (2021), no *Salmonella* was isolated from subiliac lymph nodes in cattle supplemented with or without *B. subtilis* PB6. In mice, *B. subtilis* was reported to delay colonization of and decrease *Salmonella* in lymph nodes (Mazkour et al., 2020).

Holstein steers challenged with *Salmonella typhimurium* and supplemented with *B. subtilis* PB6 for 35 d prior to the challenge had a decreased febrile response and greater white blood cell count than non-supplemented control steers during the challenge (Broadway et al., 2020). These results indicate a milder infection and stronger immune response for *B. subtilis* PB6 supplemented calves than in control. Broadway et al. (2020) also reported the calves supplemented with *B. subtilis* PB6 had a decrease in *Salmonella* colonization in small intestinal tissues and had increased BW at the end of the 35-d supplementation period compared with non-supplemented control steers. Additionally, in vitro data has demonstrated the efficacy of *B. subtilis* PB6 at inhibiting growth of several strains of *Clostridium, Salmonella,* and *Escherichia coli* (Kemin, 2019; Teo and Tan, 2005). Reduced fecal Salmonella paired with the positive health outcomes observed with *B. Subtilis* PB6 supplementation in the present study could be due, in part, to a decreased enteric pathogenic load.

As the beef industry continues to endure feedyard health challenges (Peel, 2020) studying gut health may provide information to help manage disease among feedlot cattle populations. Routine environmental, dietary, and management stressors, and antibiotic therapies can compromise commensal bacterial populations of the intestinal tract. Shifting microbial communities of the intestinal epithelial tract increases the susceptibility for pathogen invasion through lowered commensal bacterial populations, reduced viscosity of the mucosal layer of the epithelium, and increased intestinal epithelium permeability (Chase and Kaushik, 2019; Jayaraman et al., 2017). Further, the integrity of the intestinal lining and viability of the enteric immune system is pivotal to the overall health of cattle, as a review by Chase, 2018, indicated that the enteric immune system is a vital component of overall immunity and serves as the initial barrier for over 90% of pathogens.

CLOSTAT is comprised of the patented strain of the active microbial *Bacillus Subtilis* PB6 that was isolated from the gastrointestinal tract of healthy chickens that survived a severe necrotic enteritis outbreak (Teo and Tan, 2005). Direct and indirect modes of action have been identified for *B. subtilis* PB6. Directly, *B. subtilis* PB6 produces bacteriocins with activity against pathogenic strains of gram-positive and gram-negative bacteria (Teo and Tan, 2005). Secondarily, *B. subtilis* PB6 has demonstrated involvement with bacterial cross-talk through the quorum-sensing pathways, indirectly inhibiting several inflammatory pathways, promoting anti-inflammatory pathways, and maintaining certain commensal bacterial populations (Kim et al., 1998; Selvam et al., 2009). The multifaceted impacts that *B. subtilis* PB6 has on intestinal health have been delineated. Intestinal populations of *Lactobacillus* spp. were greater in broilers supplemented with *B. subtilis* PB6 than in control (Teo and Tan, 2006). Studying rats as a model to evaluate interventions for irritable bowel syndrome, intestinal inflammation was decreased with *B. subtilis* PB6 supplementation (Folignè et al., 2012). Further, Goetz et al. (2022) reported that inflammatory markers were lowered for transition dairy cattle supplemented with *B. subtilis* PB6 over control. In addition, intestinal morphology was improved with *B. subtilis* PB6 supplementation in broilers as Jayaraman et al. (2017) indicated that the villus height and crypt depth of jejunal samples, and the ratio of villus height to crypt depth in duodenal samples was greater for *B. subtilis* PB6 supplemented birds than control or birds treated with antibiotics. Jayaraman et al. (2013) also reported the villus height:crypt depth was greater for *B. subtilis* PB6 supplemented broilers during a necrotic enteritis challenge compared with control.

## CONCLUSIONS

In a large-pen, commercial feedyard setting, supplementing CLO to feedlot steers resulted in less morbidity, a tendency for lower fecal concentration but no difference in fecal prevalence of *Salmonella,* and fewer removals than un-supplemented CON steers. *Bacillus subtilis* PB6 works through multifaceted modes to decrease enteric pathogen load and improve intestinal integrity. These results demonstrate that CLO is an effective active microbial supplement for improving the overall health of feedlot cattle.
